# Identification and Characterization of an Alphacoronavirus in *Rhinolophus sinicus* and a Betacoronavirus in *Apodemus ilex* in Yunnan, China

**DOI:** 10.3390/microorganisms12071490

**Published:** 2024-07-21

**Authors:** Qian Liu, Dan-Shu Wang, Zhong-Hao Lian, Jie Fang, Pei-Yu Han, Ye Qiu, Jun-Ying Zhao, Li-Dong Zong, Yun-Zhi Zhang, Xing-Yi Ge

**Affiliations:** 1 Hunan Provincial Key Laboratory of Medical Virology, College of Biology, Hunan University, Changsha 410012, China; liuqian2022@hnu.edu.cn (Q.L.); ds_wang@hnu.edu.cn (D.-S.W.); lianzhonghao@hnu.edu.cn (Z.-H.L.); fangjie155@hnu.edu.cn (J.F.); qiuye@hnu.edu.cn (Y.Q.); 2 Yunnan Key Laboratory of Screening and Research on Anti-Pathogenic Plant Resources from Western Yunnan, Yunnan Key Laboratory of Zoonotic Disease Cross-Border Prevention and Quarantine, Institute of Preventive Medicine, School of Public Health, Dali University, Dali 671000, China; hanpeiyu1511@gmail.com (P.-Y.H.); zhaojunying0714@163.com (J.-Y.Z.); zld2019106326@163.com (L.-D.Z.)

**Keywords:** CoV, HKU2, HKU24, bats and rodents, phylogenetic analysis

## Abstract

Coronaviruses (CoVs), the largest positive-sense RNA viruses, have caused infections in both humans and animals. The cross-species transmission of CoVs poses a serious threat to public health. Rodents and bats, the two largest orders of mammals, serve as significant natural reservoirs for CoVs. It is important to monitor the CoVs carried by bats and rodents. In this study, we collected 410 fecal samples from bats and 74 intestinal samples from rats in Yunnan Province, China. Using RT-PCR, we identified one positive sample for alphacoronavirus (TC-14) from *Rhinolophus sinicus* (Chinese rufous horseshoe bat) and two positive samples for betacoronavirus (GS-53, GS-56) from *Apodemus ilex* (Rodentia: Muridae). We successfully characterized the complete genomes of TC-14 and GS-56. Phylogenetic analysis revealed that TC-14 clustered with bat CoV HKU2 and SADS-CoV, while GS-56 was closely related to rat CoV HKU24. The identification of positive selection sites and estimation of divergence dates further helped characterize the genetic evolution of TC-14 and GS-56. In summary, this research reveals the genetic evolution characteristics of TC-14 and GS-56, providing valuable references for the study of CoVs carried by bats and rodents in Yunnan Province.

## 1. Introduction

Coronaviruses (CoVs) are enveloped particles with a diameter of about 120 nm. CoV has a single-stranded positive RNA genome of approximately 30 kb in length [1]. According to the classification criteria of the International Committee on Taxonomy of Virus (ICTV), CoVs belong to the subfamily *Orthocoronavirinae* of the family *Coronaviridae* in the order *Nidovirales*. They are classified into four genera: *Alphacoronavirus* (*α-CoV*), *Betacoronavirus* (*β-CoV*), *Gammacoronavirus* (*γ-CoV*), and *Deltacoronavirus* (*δ-CoV*) [2]. In general, α-CoVs and β-CoVs infect mammals, while γ-CoVs and δ-CoVs mostly infect birds. However, a few γ-CoVs and δ-CoVs can also infect mammals [3]. Among the α-CoVs and β-CoVs, eight are known to infect humans, including severe acute respiratory syndrome-related coronavirus (SARS-CoV), SARS-CoV-2, Middle East respiratory syndrome-related coronavirus (MERS-CoV), human coronavirus 229E (HCoV-299E), human coronavirus OC43 (HCoV-OC43), human coronavirus NL63 (HCoV-NL63), human coronavirus HKU1 (HCoV-HKU1), and human–porcine deltacoronavirus CoV (Hu-PDCoV) [4,5,6]. SARS-CoV, SARS-CoV-2, and MERS-CoV are highly pathogenic human CoVs, which are all β-CoVs. SARS-CoV emerged and circulated in 2002~2003, MERS-CoV emerged in 2012, and SARS-CoV-2 emerged at the end of 2019. Currently, SARS-CoV-2 is circulating worldwide, and its pandemic seriously endangers human health [7,8,9]. Additionally, some α-CoVs and β-CoVs have also caused significant diseases in livestock, such as porcine transmissible gastroenteritis virus (TGEV), porcine respiratory coronavirus (PRCV), porcine epidemic diarrhea virus (PEDV), and swine acute diarrhea syndrome coronavirus (SADS-CoV, also known as PEAV) [10,11,12].

Previous research indicates that bats (Chiroptera) and rodents (Rodentia) are the primary hosts of CoVs that affect humans. For instance, bats naturally carry the prototype strains of SARS-CoV, MERS-CoV, SARS-CoV-2, HCoV-229E, and HCoV-NL63, and rodents are the natural hosts of the prototype strains of HCoV-HKU1 and HCoV-OC43 [4,5]. Additionally, numerous α-CoVs and β-CoVs have been discovered in bats, leading to the hypothesis that these viruses originated from bats [13]. For example, evidence indicates that SADS-CoV resulted from cross-species transmission from bats to pigs [14,15], and highly similar viral strains to SADS-CoV have been detected in bats [16]. Moreover, there are close relationships between bat CoVs and other animal CoVs, such as bovine CoVs and canine CoVs [17]. Therefore, bats are highly likely to play a crucial role in the transmission of CoVs among mammals, underscoring the importance of monitoring the evolution of CoVs in bats.

Bats are the second largest order of mammals and are uniquely capable of flight [18]. They primarily live in tropical and subtropical regions and mainly feed on nectar, fruit, pollen, insects, and blood. Bats serve as reservoirs for a large variety of viruses due to their special immune system, diverse ecology, biology, and unique traits [19,20]. Similarly, rodents, the largest order of mammals, also play a crucial role as reservoirs for pathogens and can carry more than 68 viruses that are associated with diseases in humans [21]. Since many rodents live in close proximity to humans, there is a high risk of virus transmission to humans [22]. For example, a comprehensive evaluation of zoonotic spillover and transmission potential of wildlife-derived viruses identified several rodent-related CoVs among the top 50 [23]. Furthermore, the S1/S2 furin cleavage site is commonly detected in the spike (S) protein of rodent-associated CoVs, suggesting its potential role in zoonotic transfer [24]. Given the diversity of the species of bats and rodents, it is crucial to explore and monitor the unknown viruses they carry. This research aims to provide etiological and epidemiological support for the CoVs.

In recent years, CoVs closely related to SARS-CoV and SARS-CoV-2 have been discovered in bats [25,26]. This study aims to investigate the prevalence and new strains of CoVs in bats and rodents to enhance our understanding of the genetic diversity of CoVs and for the benefit of controlling the prevalence of related infectious diseases in the future. We collected 410 fecal samples from bats and 74 intestinal samples from rodents in Yunnan Province. Using RT-PCR, we detected one positive bat-CoV sample (TC-14) belonging to the *α-CoV*, and two positive rodent-CoV samples (GS-53, GS-56) belonging to the *β-CoV*. Subsequently, we obtained the complete genome sequences of TC-14 and GS-56. Finally, we performed a phylogenetic analysis to compare their evolutionary characteristics with other CoVs, estimated their divergence time, and tested the selection pressure of codon sites.

## 2. Materials and Methods

### 2.1. Ethics Statement

The collection of small animals was performed by veterinarians with approval from the Animal Ethics Committee of Dali University (DLDXLL2020007).

### 2.2. Samples Collection and Host Identification

From August 2022 to October 2022, a total of 410 fecal samples of bats (from Menghai and Tengchong) and 74 intestinal samples of rodents (Gongshan) were collected in Yunnan Province, China. For bats, without disturbing and destroying the habitat environment of bats, only bat feces samples in caves were taken with sterilized cotton swabs. Bat samples were placed in a virus transport medium (VTM) and kept in dry ice. They were stored at −80 °C after being transported to the laboratory. For rodents, after being trapped using food, they were brought back to the laboratory in a timely manner and euthanized for dissection, and the rectal tissue at the end of the large intestine was collected. The host species was identified by morphological identification and NADH dehydrogenase subunit I (ND1) gene of the Mitochondria DNA (mtDNA) sequencing. PCR was performed based on the corresponding ND1 primers (F: CCTCGATGTTGGATCAGG, R: GTATGGGCCCGATAGCTT) [27]. The ND1 gene fragments were amplified using the DNA extracted from bat and rodent samples, then sequenced and used the sequences to BLAST against GenBank data to determine host species. Bats in Menghai and Tengchong were identified as *Hipposideros armiger* and *Rhinolophus sinicus*, respectively. Rodents in Gongshan were identified as *Apodemus ilex*.

### 2.3. RNA Extraction and CoVs RT-PCR Screening

For the fecal samples, the automated nucleic acid extractor (Bioer, Hangzhou, China) was used to extract RNA. The intestinal samples were ground and centrifuged, and 200 μL of the supernatant was used for RNA extraction. Then, the nucleic acid was stored at −80 °C as the template for RT-PCR. Initial CoV detection was performed by amplifying a 434 bp fragment of the RNA-dependent RNA polymerase (RdRp) sequence of CoVs using CoV conserved primers, FWD3 (5′-GGTTGGGAYTAYCCHAARTGTGA-3′), FWD4 (5′-GAYTAYCCHAARTGTGAUMGWGC-3′), and RVS3 (5′-CCATCATCASWYRAATCATCATA-3′), as described previously [28]. The nested PCR amplification was performed using PrimeScript™ One-Step RT-PCR Kit Ver.2 (Takara, Beijing, China) and Ex Taq^®^ DNA polymerase (Takara, Beijing, China). The first round used a One-Step RT-PCR kit on 20 μL PCR mixture including 0.8 μL PrimeScript 1 step enzyme, 10 μL 2 × 1 step buffer, 1 μL FWD3 primer (0.5 μM), 1 μL RVS3 primer (0.5 μM), 5.2 μL ddH_2_O, and 2 μL extracted RNA. The mixtures were amplified for 40 cycles at 50 °C for 30 min, 94 °C for 2 min, 94 °C for 30 s, 50 °C for 30 s, and 72 °C for 40 s, and a final extension at 72 °C for 10 min. The second round used Ex Taq DNA polymerase kit on 25 μL PCR mixture including 0.25 μL Ex Taq enzyme (25 U), 10 μL 10 × Ex Taq Buffer, 2 μL dNTP mixture (0.2 μM), 1 μL FWD4 primer (0.5 μM), 1 μL RVS3 primer (0.5 μM), 16.25 μL ddH_2_O, and 2 μL product of first round. The mixtures were amplified for 35 cycles as 98 °C for 3 min, 98 °C for 10 s, 50 °C for 30 s, and 72 °C for 20 s, and a final extension at 72 °C for 5 min. The PCR product was sent to the company (Tsingke, Beijing, China) for sequencing; then, the result was compared with known RdRp sequences of CoVs in the GenBank database and the complete genomes with high identity were selected as reference sequences.

### 2.4. Complete Genome Sequencing

The complete genome of TC-14 and GS-56 were amplified using extracted RNA as a template. RNA was amplified with degenerate primers, which were designed by multiple alignments of reference complete genomes using the PrimeScript™ One-Step RT-PCR kit version 2 (Takara, Beijing, China). Additional primers were designed according to the results of the first and subsequent rounds. The 5′ and 3′ genome end sequences were obtained by T-A cloning. The sequences were assembled to obtain the full-length genome sequence (Table S4).

### 2.5. Genomic and Phylogenetic Analysis

Multiple sequence alignment was performed by MAFFT v7.149 in BioAider v1.527 [29,30]. The maximum likelihood phylogenetic tree of the RdRp domain was performed by IQ-tree v2.1.3 with 10,000 ultrafast bootstraps, and the most appropriate model was calculated using the ModelFinder program according to the corrected akaike information criterion method, and the most appropriate substitute model was LG + F + I + G4 [31,32].

### 2.6. Identification of Positive Selection Sites

To detect positive selection sites within α-CoVs and β-CoVs, the codeml program in the PAML v4.9 package was used for the selection pressure analysis [33]. Three-pair site models in PAML were applied, including M0–M3, M1a–M2a, and M7–M8. The M0, M1a, and M7 are null hypothesis models, and the M3, M2a, and M8 are corresponding alternative models, respectively. Then, the likelihood ratio test (LRT) was used to test the significance of the differences between paired models in jModelTest v2.1.6 software, and a significance level of 0.05 was applied [34,35]. Finally, the Bayes empirical Bayes (BEB) was used to calculate the posterior probabilities of positive selection sites [36].

### 2.7. Estimation of Divergence Dates

The *RdRp* sequences were aligned using the MAFFT v7.149 program with the codon method in BioAider v1.527 [29,30]. Then, the ModelFinder was used to estimate the substitution model with Bayesian information criteria (BIC), and the most appropriate substitute model of nucleotide was GTR + F + I + G4 [37]. Next, BEAST v1.10.4 software was used to infer divergence time, and a Markov chain of 20 million steps with sampling every 2000 steps was used in BEAST [38]. The calibrations of divergence times (ya, years ago) were: alphacoronavirus (mean of 2850 ya and SD of 700 ya), betacoronavirus (mean of 3440 ya and SD of 800 ya), HKU2-related lineage (mean of 240 ya and SD of 100 ya), and MERS-CoVs and HKU4/5 (mean of 840 ya and SD of 300 ya) with normal distribution, referring to previous reports [39]. The prior mean evolution rate was 1 × 10^−4^~1 × 10^−3^ subs/site/year with uniform distribution, and the time of the most recent common ancestor (tMRCA) was calculated under an uncorrelated lognormal relaxed clock. The Tracer v1.7 program was used to check the Effective Sample Size (ESS) of all estimated parameters and ensured each ESS was greater than 200 to achieve convergence [40]. Finally, the Maximum Clade Credibility (MCC) tree was obtained by discarding the first 10% of states in the Tree Annotator v1.10.4 package.

## 3. Results

### 3.1. Identification of Bat and Rodent CoVs

**Figure 1:**
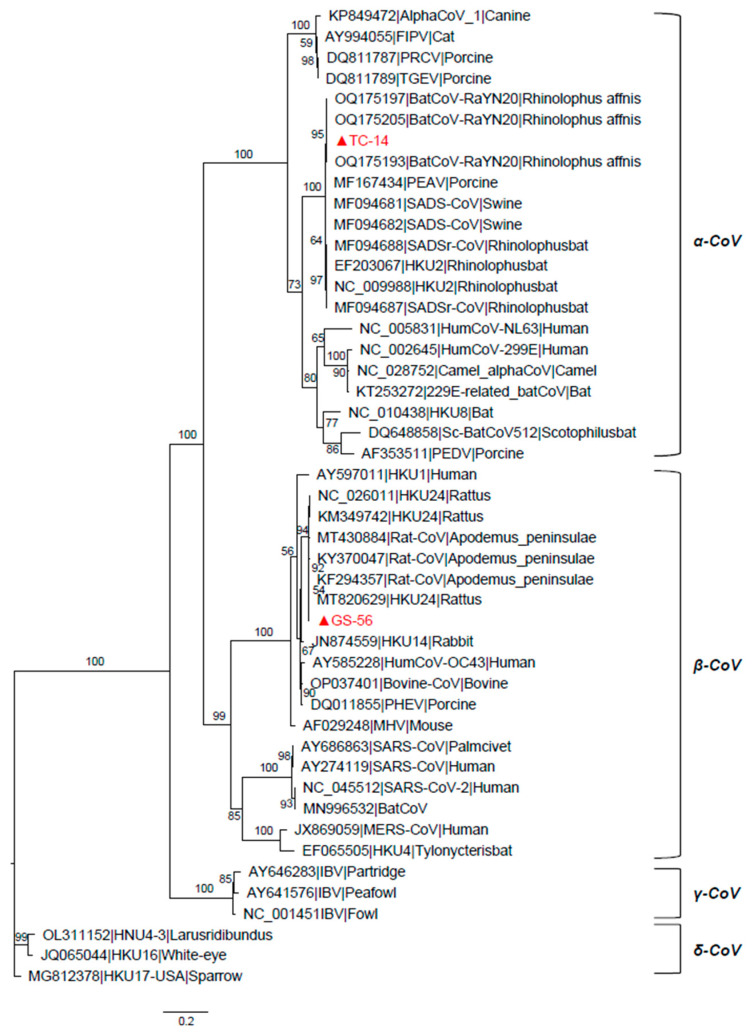
The maximum likelihood tree of RNA-dependent RNA polymerase (RdRp) sequences of TC-14, GS-56, and other CoVs. The tree was constructed using IQ-tree with LG + F + I + G4 substitution model and 10,000 ultrafast bootstraps. The scale bar indicates amino acid substitution per site, and four genera of coronavirus are marked on the side. The TC-14 and GS-56 are marked in red and with a triangle.

A total of 344 fecal samples of *R. sinicus* (Chinese rufous horseshoe bat) were collected in Tengchong, along with 66 fecal samples of *Hipposideros armiger* (great roundleaf bat) in Menghai, and 74 intestinal samples of *A. ilex* (Rodentia: Muridae) in Gongshan, Yunnan Province (Table 1). By amplifying the 434 nt *RdRp* fragment of CoV, we identified one CoV-positive sample named TC-14 in fecal samples of *R. sinicus* and two CoV-positive samples in intestinal samples of *A. ilex* named GS-53 and GS-56. Since GS-53 and GS-56 shared 100% sequence identity in the spike (S) gene and nucleocapsid (N) gene, we only amplified the whole genome of GS-56. The preliminary phylogenetic analysis based on *RdRp* sequences confirmed that TC-14 belonged to the *α-CoV* and GS-56 belonged to the *β-CoV* (Figure 1).

### 3.2. Genome Analysis of TC-14 and GS-56

To further reveal the genomic characteristics of TC-14 and GS-56, we annotated their genome structure based on two highly similar genomic sequences. The complete genome of TC-14 is 27,173 nt in length, containing the following regions: 5′ untranslated region (5′-UTR), open reading frame (ORF) 1ab, spike (S), non-structural (NS) 3, envelope (E), membrane (M), nucleocapsid (N), 7a, and 3′ untranslated region (3′-UTR). For GS-56, the genome size is 31,273 nt; besides the 5′-UTR and 3′-UTR, we identified 10 typical ORFs, including ORF1ab, NS2a, hemagglutinin esterase (HE), S, NS4, NS5, E, M, N2, and N (Table 2 and Supplementary Figure S1).

Through genomic and gene sequence alignment and comparative analysis, it was determined that the complete genomic sequence of TC-14 shared 97.57% nucleotide (nt) identity with BtRa-AlphaCoV/YN2020 (OQ175205, *Rhinolophus affinis*) and 93.54% nt identity with SADSr-CoV (MF094687, *Rhinolophus* sp.). Similarly, the GS-56 complete genome shared 93.68% nt identity with RtAp-CoV/Tibet2014 (KY370047, *Apodemus peninsulae*) and 92.98% nt identity with ChRCoV HKU24 (MT820630, *Apodemus agrarius*). In addition, the S protein of TC-14 had 94.43% amino acid (aa) identity with SADSr-CoV (MF094687, *Rhinolophus* sp.) and 93.98% aa identity with SADS-CoV (MF094684, swine). The high aa identity of the S protein among TC-14, SADS-CoV, and SADSr-CoV suggests a close phylogenetic relationship and the potential for cross-species transmission. The S protein of GS-56 shared 96.02% aa identity with ChRCoV HKU24 (MT820629, *Apodemus chevrieri*). Furthermore, the aa identity among TC-14, GS-56, and other CoVs in ORFs is shown in Supplementary Table S1.

Given that ORF1ab is the largest ORF in the CoV genome and can be cleaved into 16 non-structural proteins (NSPs) by the papain-like protease (PLpro) and 3C-like protease (3CLpro), we then predicted the cleavage sites of NSPs for TC-14 and GS-56. In summary, we did not identify any new types of cleavage site, but we found that the NSP1/NSP2 cleavage site of GS-56 was the same as that of ChRCoV HKU24, which is unique in the *China Rattus coronavirus HKU24* species (Supplementary Table S2). To confirm the taxonomy of TC-14 and GS-56, we further compared the aa identity of five conserved replicase domains (3CLpro, NiRAN, RdRp, ZBD, and HEL1) between TC-14 and RhHKU2, and between GS-56 and ChRCoV HKU24, respectively. The results showed that TC-14 could be classified as the *Rhinolophus bat coronavirus HKU2* species, and GS-56 could be classified as the *China Rattus* *coronavirus HKU24* species, as the aa identity between them was over 95% in the five conserved replicase domains (Supplementary Table S3).

### 3.3. Phylogenetic Analyses of the TC-14 and GS-56

**Figure 2:**
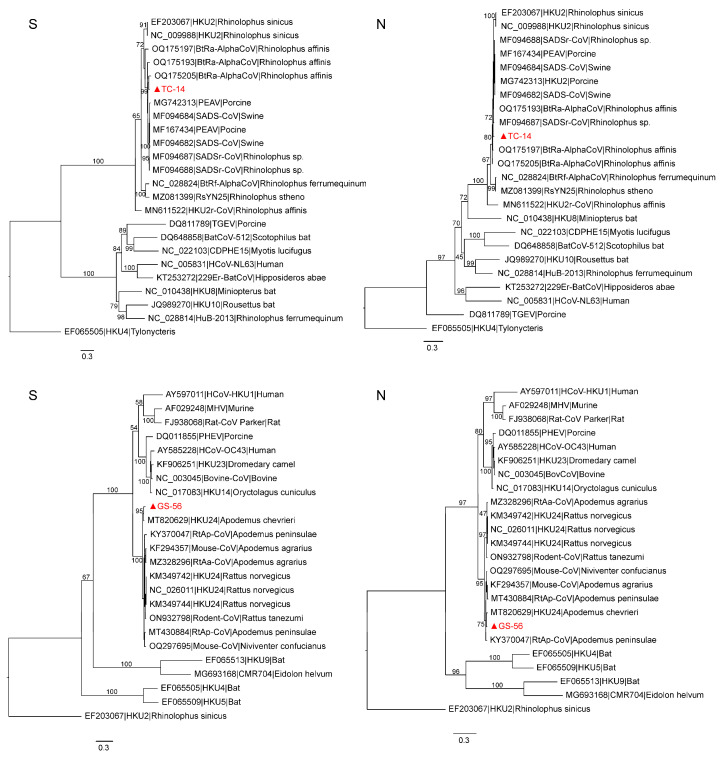
Phylogenetic trees of amino acid sequences of S and N proteins of TC-14 and GS-56 CoVs. These trees were constructed using IQ-tree with 10,000 ultrafast bootstraps, and their substitution models are as follows: WAG + F + I + G4, pfam + F + I + G4, WAG + F + G4, pfam + F + G4, respectively. The accession number, taxonomy, and host of each sequence are displayed. TC-14 and GS-56 are marked in red and with a triangle.

**Figure 3:**
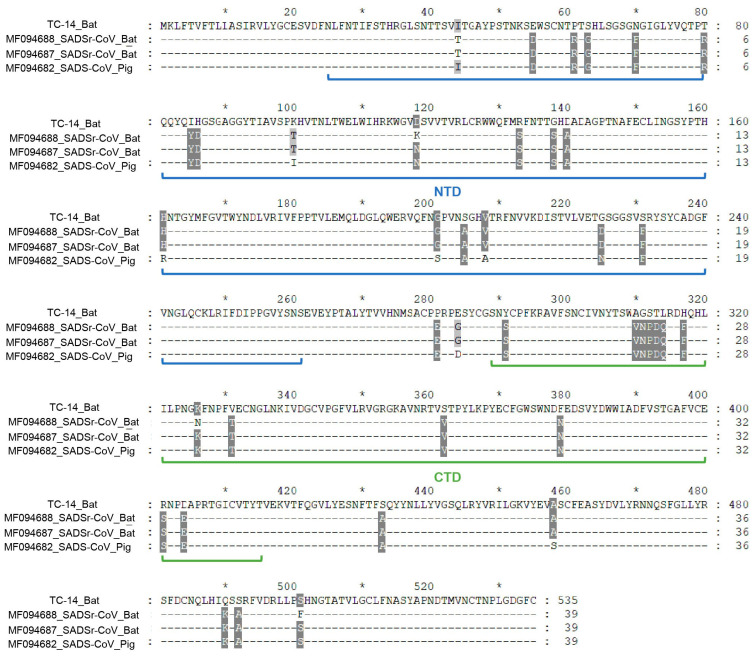
Multiple alignments of S1 region of TC-14, SADS-CoV, and SADSr-CoV. The accession number and host of the selective sequences are shown. The annotation of the NTD domain in S1 is with reference to SADS-CoV; the NTD domain is marked with a blue line, and the CTD domain is marked with a green line. The short horizontal line indicated the identical sites. * indicates odd multiples of ten of the amino acid.

**Figure 4:**
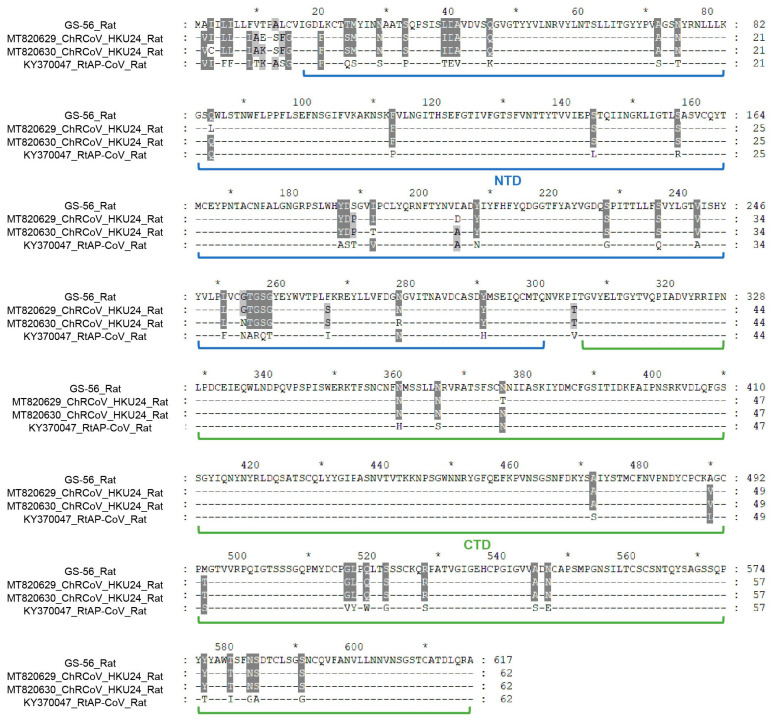
Multiple alignment of S1 region of GS-56, ChRCoV-HKU24, and RtAp-CoV. The accession number and host of the genomes are shown. The annotation of NTD and CTD domains in S1 is a reference to ChRCoV HKU24. The NTD domain is marked with a blue line, and the CTD domain is marked with a green line. The short horizontal line indicates the identical sites. * indicates odd multiples of ten of the amino acid.

In order to explore the deep evolutionary relationship, we constructed phylogenetic trees of the ORF1ab, S, and N proteins based on the amino acid sequences of TC-14, GS-56, and other CoVs. The results showed that the three critical ORFs of TC-14 were clustered together with BtRa-AlphaCoV/YN2020 and grouped into the RhHKU2-related clade (Figure 2 and Supplementary Figure S2). For GS-56, the ORF1ab and S proteins were clustered with ChRCoV HKU24, with more than 95% amino acid identity. But, for the N protein, GS-56 had a closer phylogenetic relationship with RtAp-CoV/Tibet2014 compared to ChRCoV HKU24 (Figure 2 and Supplementary Figure S2). Next, we compared the S1 region of TC-14 and GS-56 with SADSr-CoV and ChRCoV HKU24, respectively. The multiple alignment of TC-14, SADSr-CoV, and SADS-CoV showed that there were 39 aa mutations in the S1 region (Figure 3). For GS-56, ChRCoV HKU24, and RtAp-CoV, there were 62 aa mutations in the S1 region (Figure 4).

### 3.4. Positive Selection Analysis

To identify positive selection sites in α-CoVs and β-CoVs, including TC-14 and GS-56, we conducted selective selection pressure analysis in ORF1ab, S, E, M, and N. In summary, significant differences between the M7 and M8 models were only found in ORF1ab and S, as determined by the LRT method. In α-CoVs, a total of 12 possible positive selection sites were detected in the S gene, and 10 possible positive selection sites were detected in the ORF1ab. In β-CoVs, two possible positive selection sites were detected in the S gene, and three possible positive selection sites were detected in the ORF1ab. Among these selection sites, three sites (133R, 138G, 312S) in the S protein and four sites (112N, 161R, 423S, 2292V) in the ORF1ab protein from α-CoVs showed significant differences (Table 3). In the S protein, the aa residues 133R and 138G were located in the NTD domain, while 312S was located in the CTD domain. The three significant positive selection sites of S protein in α-CoVs were visualized by Modeller (Supplementary Figure S3). Since the NTD is responsible for binding to the host receptor, these three positive selection sites may have functional implications for viral invasion. Additionally, considering that the ORF1ab protein played a crucial role in viral transcription and replication, the significant positive selection sites detected in ORF1ab may affect the process of virion genomic replication and gene expression.

### 3.5. Estimation of Divergence Dates

**Figure 5:**
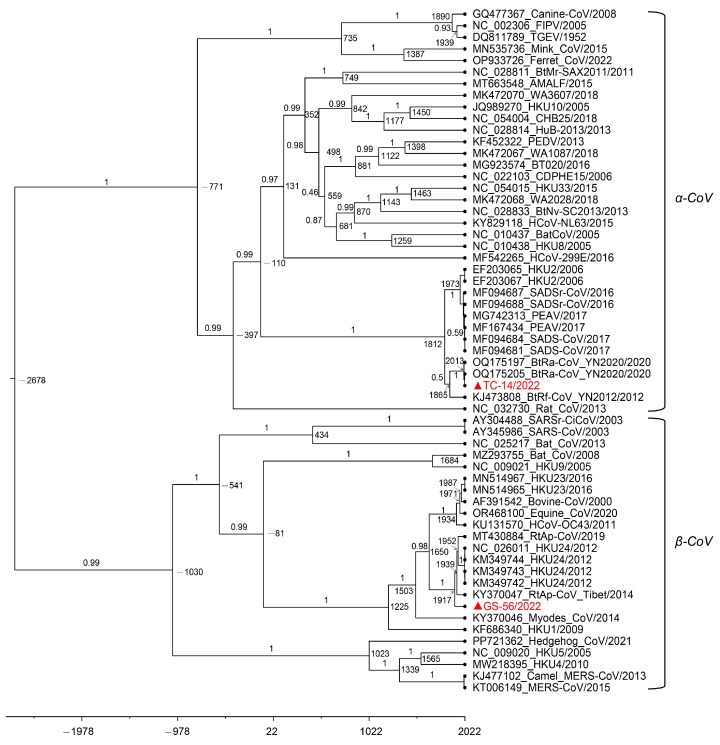
Maximum clade credibility (MCC) tree with divergence time based on *RdRp* nucleotide sequence. The substitution model was GTR + F + F + I + G4, the tree was obtained by Model Finder under the BIC standard [37]. The value near the node indicates the age of the node, and the label on the branch represents the Bayesian posterior probability. The arrows are used to accurately locate points of divergence. The species and sampling time of the selected sequences are labeled. The TC-14 and GS-56 are marked in red.

We estimated the divergence dates of TC-14 and GS-56 by analyzing the *RdRp* nucleotide sequences of α-CoVs and β-CoVs (Figure 5). The mean evolutionary rate of α-CoVs and β-CoVs was approximately 2.58 × 10^−4^ subs/site/year (95% highest posterior density intervals or HPDs, 1.68 × 10^−4^–3.60 × 10^−4^). Using the molecular clock dating with the *RdRp* gene, we determined that the tMRCA of *α-CoV* was around 750 B.C. (1766 B.C.–113 A.D., 95% HPD), the tMRCA of *β-CoV* was around 1086 B.C. (2250 B.C.–10 A.D., 95% HPD), and the tMRCA of the HKU2-related lineage was approximately 1821 A.D. (1710A.D–1902 A.D., 95% HPD). Additionally, the tMRCA of TC-14 and BtRa-CoV_YN2020 was estimated to be around 2013 A.D. (2002 A.D.–2020 A.D., 95% HPD), and the tMRCA of GS-56 was approximately 1920 A.D. (1860 A.D.–1964 A.D., 95% HPD). Furthermore, based on its divergence time, we found that GS-56 had relatively old evolutionary relationships within the HKU24-related lineage.

## 4. Discussion

The cross-species transmission of CoVs has caused a number of infections in both animals and humans. For example, human CoVs, including SARS-CoV, MERS-CoV, and SARS-CoV-2, can infect humans and cause serious diseases [41,42]. The outbreak of SARS-CoV-2 in late 2019 led to a global pandemic and seriously damaged human health. Additionally, CoVs exhibit a high level of genetic diversity and have been found in nearly all known mammals [17,43]. Rodents and bats, in particular, which are the largest and second-largest orders of mammals, respectively, serve as critical natural reservoirs for CoVs. Among the four genera of CoVs, a substantial number of α-CoVs and β-CoVs have been identified in bats and rodents. Studies have also demonstrated that over 70% of CoV-related infectious diseases in humans are associated with animal CoVs [7,13].

Since the outbreak of SARS in 2003, it has been discovered that *R. sinicus* carries a diversity of SARS-like coronavirus [25,44]. A bat CoV known as RaTG13 (MN996532) was found in *R. sinicus* and showed 96.2% identity to SARS-CoV-2 at the complete genome level [26]. Additionally, various α-CoVs and β-CoVs have been detected in rodents like *Apodemus chevrieri* and *Eothenomys miletus* [45]. These findings emphasize the importance of monitoring the presence of CoVs in both bats and rodents.

In this study, we obtained the complete genomes of an α-CoV (TC-14) from *R. sinicus* and a β-CoV from *A. ilex.* Phylogenetic analysis revealed that TC-14 is closely related to HKU2, while GS-56 is closely related to HKU24. Previous research has shown that the fatal SADS in Guangdong Province was caused by an SADSr-CoV of bat origin [14]. We detected a highly similar strain in Yunnan bat samples with a complete genome identity of 92.8% between TC-14 and SADSr-CoV. This highlights the importance of monitoring bat coronavirus to prevent the occurrence of similar epidemics.

Additionally, studies have suggested that a ChRCoV HKU24 sequence (KM349742) may be the source of recombination in the S region of SARS-CoV-2 [40,46]. Furthermore, GS-56 shares more than 85% identity with the S gene of HKU24, highlighting the importance of monitoring rodent coronaviruses. CoVs infect hosts by using their S protein to recognize the host receptor [47]. Our identification of positive selection sites on the S gene and ORF1ab of α-CoVs suggests that these sites may play a critical role in viral replication and entry. Lastly, we estimated the divergence date of TC-14 and GS-56 with other CoVs and found that GS-56 has relatively old evolutionary relationships within the HKU24-related lineage.

Zoonotic viruses that originate from wildlife reservoirs cause emerging infectious diseases occasionally. Unfortunately, the existence and prevalence of wildlife viruses have not yet been fully revealed. CoV infects various mammals and shows high genetic diversity and cross-species transmission potential. Here, this study detected new strains of an α-CoV in *R. sinicus* and a β-CoV GS-56 in *A. ilex* in Yunnan Province, expanding our understanding of the geographical distribution and host of these viruses. The results further revealed the genetic evolution characteristics of TC-14 and GS-56, further enriching the information on CoVs carried by bats and rodents in Yunnan Province. These findings may contribute to the prevention and control of the spread and pathogenicity of related CoVs.

## Figures and Tables

**Table 1:** Detection results of bat and rodent coronavirus. MH—Menghai, Yunnan province; TC—Tengchong, Yunnan province; GS—Gongshan, Yunnan province.

Samples Type	Sampling Location	Sample Host	Samples	Positive Samples	Positive Rate (%)
Bat feces	MH	*Hipposideros armiger*	66	0	0
	TC	*Rhinolophus sinicus*	344	1 (TC-14)	0.29
Rat intestine	GS	*Apodemus ilex*	74	2 (GS-53, GS-56)	2.7

**Table 2:** The coding potentials of TC-14 and GS-56 genomes.

CoV	ORF	Start-End (Nucleotide Position)	Length (nt)	Length (aa)
TC-14	1ab	304–20,489	20,186	6728
	S	20,486–23,878	3393	1131
	NS3	23,878–24,567	690	230
	E	24,548–24,775	228	76
	M	24,784–25,470	687	229
	N	25,482–26,609	1128	376
	NS7a	26,621–26,932	312	104
GS-56	1ab	195–21,661	21,467	7155
	NS2a	21,663–22,493	831	277
	HE	22,508–23,782	1275	425
	S	23,798–27,874	4077	1359
	NS4	27,967–28,380	414	138
	NS5	28,362–28,676	315	105
	E	28,669–28,917	249	83
	M	28,932–29,627	696	232
	N2	29,620–30,312	693	231
	N	29,637–30,968	1332	444

**Table 3:** The results of the positive selection sites in α-CoVs and β-CoVs by PAML.

CoVs	Gene	ComparedModels	Positive Selected Sites (BEB Results, *: *p* > 95%, **: *p* > 99%)
α-CoV	S	M7–M8	80T, 86H, 114K, 133R *, 138G *, 281P, 311G, 312S *, 313T, 314L, 331V, 338K
	ORF1ab	M7–M8	112N **, 161R *, 423S **, 450L, 533A, 632V, 1390S, 433T, 2292V *, 3128A
β-CoV	S	M7–M8	68S, 364S
	ORF1ab	M7–M8	269T, 1234L, 1475L

## Data Availability

The datasets analyzed during the current study are available from the corresponding authors upon reasonable request. All the sequences in this manuscript can be obtained from the NCBI database GenBank with the following accession numbers: PP974703 and PP974704 (https://www.ncbi.nlm.nih.gov, accessed on 2 July 2024).

## Supplementary Materials

The following supporting information can be downloaded at: https://www.mdpi.com/article/10.3390/microorganisms12071490/s1, Figure S1: The genome structure of TC-14 and GS-56 were predicted by referring to two sequences with high identity; Figure S2: The maximum likelihood tree of amino acid sequences of TC-14 ORF1ab and GS-56 ORF1ab protein. Figure S3: Visualization of three significant positive selection sites in α-CoV. Table S1: The amino acid identity of ORFs among TC-14, GS-56, and other CoVs; Table S2: The NSPs cleavage sites prediction of TC-14 and GS-56; Table S3: The amino acid identities of five conserved domains between TC-14 and HKU2-related CoVs, GS-56 and HKU24-related CoVs. Table S4: The sequences of the degenerated sequences that were used for complete genome sequencing.
