# Supplementary material for: Identification and Characterization of an Alphacoronavirus in Rhinolophus sinicus and a Betacoronavirus in Apodemus ilex in Yunnan, China
Source: Microorganisms. 2024 Jul 21;12(7):1490. doi: 10.3390/microorganisms12071490 (PMC11278907; doi:10.3390/microorganisms12071490)
Supplement: Supplementary file 1 [file microorganisms-12-01490-s001.zip › Supplementary Figure S1.pdf]

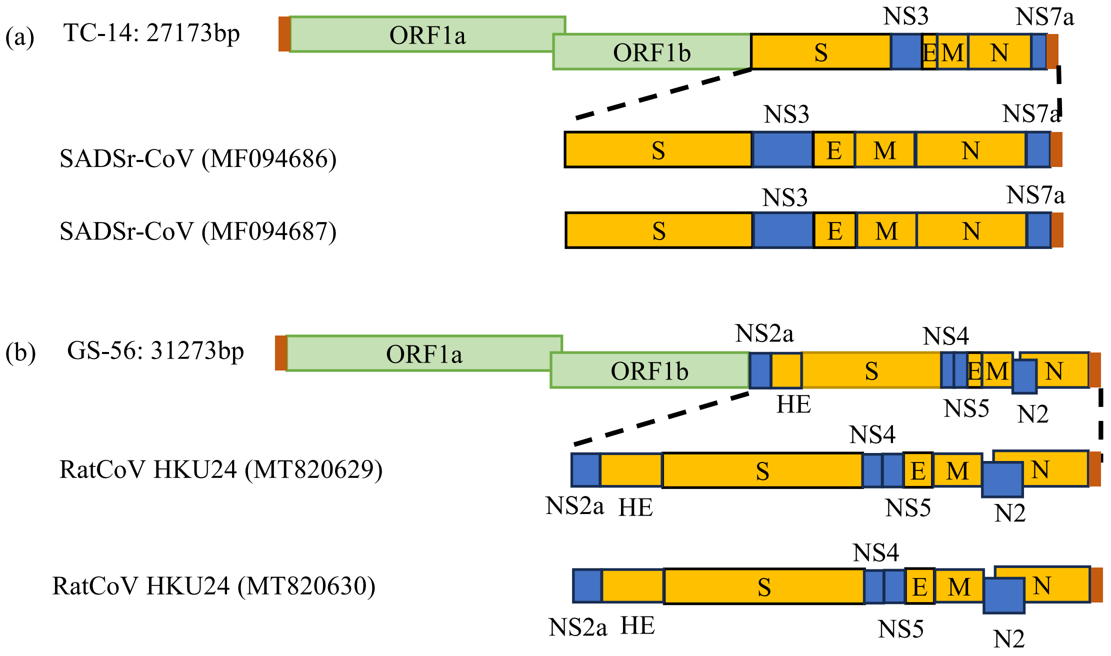

Supplementary Figure S1: The genome structure of TC-14 and GS-56 were predicted by referring to two sequences with high identity. Green, polyproteins ORF1a and ORF1b; Yellow, structure proteins ; Blue, accessory proteins; Orange, untranslated regions.
