# Supplementary material for: Identification and Characterization of an Alphacoronavirus in Rhinolophus sinicus and a Betacoronavirus in Apodemus ilex in Yunnan, China
Source: Microorganisms. 2024 Jul 21;12(7):1490. doi: 10.3390/microorganisms12071490 (PMC11278907; doi:10.3390/microorganisms12071490)
Supplement: Supplementary file 1 [file microorganisms-12-01490-s001.zip › Supplementary Figure S2.pdf]

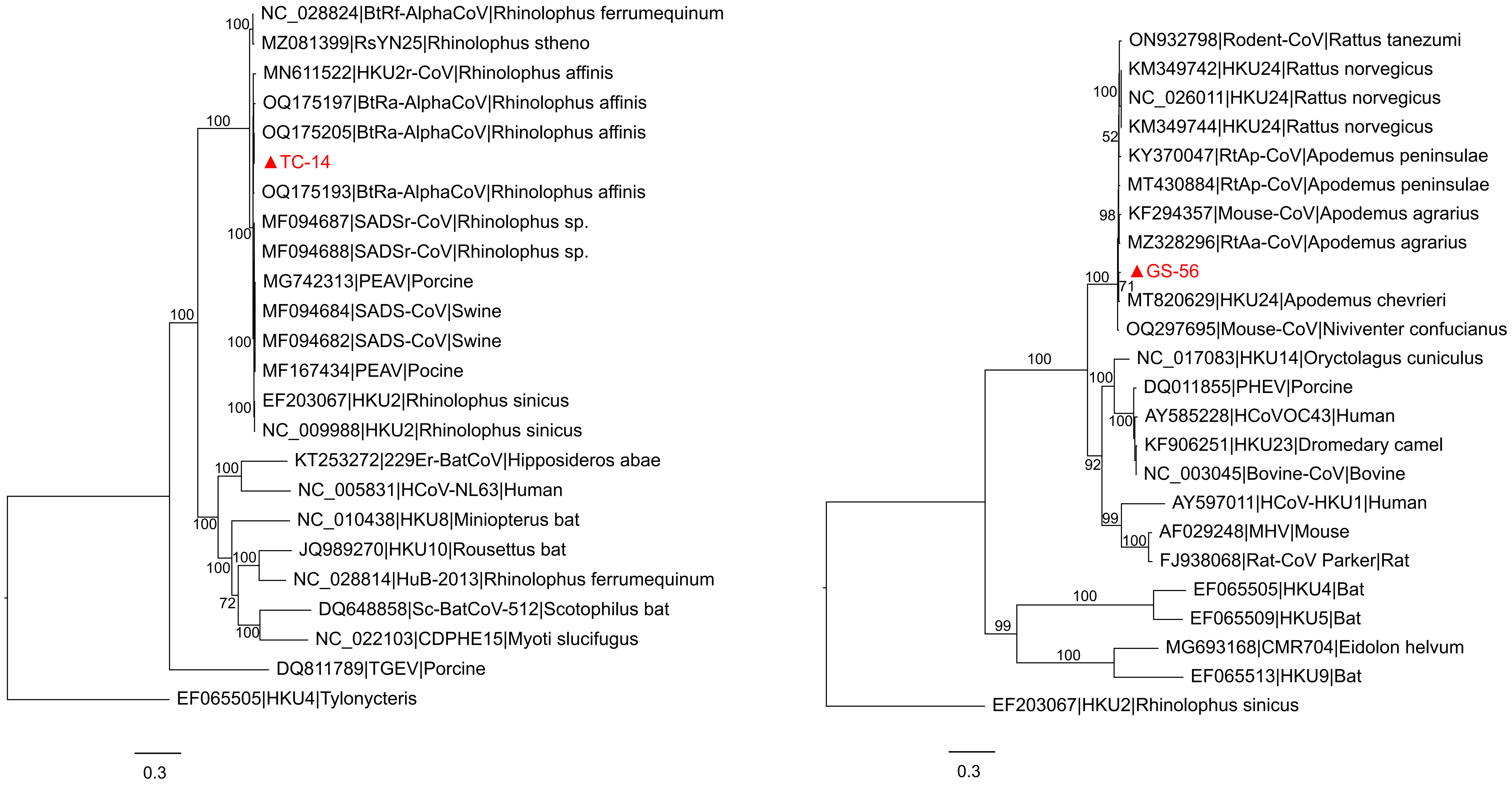

Supplementary Figure S2: The maximum likelihood tree of amino acid sequences of TC-14 ORF1ab and GS-56 ORF1ab protein. These trees were constructed using IQ-tree with 10,000 ultrafast bootstraps, and their substitution models were LG+F+I+G4. TC-14 and GS-56 were marked in red and triangle.
