# Supplementary material for: Identification and Characterization of an Alphacoronavirus in Rhinolophus sinicus and a Betacoronavirus in Apodemus ilex in Yunnan, China
Source: Microorganisms. 2024 Jul 21;12(7):1490. doi: 10.3390/microorganisms12071490 (PMC11278907; doi:10.3390/microorganisms12071490)
Supplement: Supplementary file 1 [file microorganisms-12-01490-s001.zip › Supplementary Table S1.pdf]

Supplementary Table S1: The amino acid identity of ORFs among TC-14, GS-56 and other CoVs.

CoV(id)	TC-14 ORFs pairwise amino acid identity (%)						
	1ab	S	NS3	E	M	N	NS7a
OQ175205|BatCoV-RaYN20	99.36	93.31	98.69	100	100	97.33	62.62
OQ175197|BatCoV-RaYN20	98.30	85.77	97.81	98.66	98.68	97.60	98.73
MF094687|SADSr-CoV	98.27	94.43	95.63	100	97.36	96.80	95.14
MF094684|SADS-CoV	98.14	93.98	94.76	100	98.24	95.73	60.60
MG742313|PEAV	98.11	93.98	94.76	97.33	96.93	95.73	60.60
EF203067|HKU2	97.86	86.28	96.50	98.66	98.24	94.40	57.57
NC_010438|HKU8	59.39	22.00	40.61	48.64	64.13	48.24	12.60-

CoV(id)	GS-56 ORFs pairwise amino acid identity (%)							
	1ab	NS2a	S	E	M	NS4	NS5	N	N2
KY370047|Rodent-CoV	97.46	85.31	92.34	98.78	99.56	88.64	88.57	96.17	-
OQ297695|RaNc-CoV	97.72	81.90	91.75	100	99.13	89.05	96.19	96.84	-
MT820630|HKU24	97.69	83.33	90.66	98.78	99.13	86.86	89.42	93.45	100
MT820629|HKU24	97.03	90.21	96.02	98.78	98.26	89.05	90.38	97.29	76.52
NC_026011|HKU24	97.16	84.24	91.31	97.56	99.13	86.76	89.42	93.45	-
LC061272| Equine-CoV	73.68	54.83	69.17	76.82	84.78	-	14.28	72.46	-

CoV(id)	GS-56 ORFs pairwise amino acid identity (%)								
	1ab	NS2a	S	E	M	NS4	NS5	N	N2
KY370047|Rodent-CoV	97.46	85.31	92.34	98.78	99.56	88.64	88.57	96.17	-
OQ297695|RaNc-CoV	97.72	81.90	91.75	100	99.13	89.05	96.19	96.84	-
MT820630|HKU24	97.69	83.33	90.66	98.78	99.13	86.86	89.42	93.45	100
MT820629|HKU24	97.03	90.21	96.02	98.78	98.26	89.05	90.38	97.29	76.52
NC_026011|HKU24	97.16	84.24	91.31	97.56	99.13	86.76	89.42	93.45	-
LC061272| Equine-CoV	73.68	54.83	69.17	76.82	84.78	-	14.28	72.46	-
