# Supplementary material for: Identification and Characterization of an Alphacoronavirus in Rhinolophus sinicus and a Betacoronavirus in Apodemus ilex in Yunnan, China
Source: Microorganisms. 2024 Jul 21;12(7):1490. doi: 10.3390/microorganisms12071490 (PMC11278907; doi:10.3390/microorganisms12071490)
Supplement: Supplementary file 1 [file microorganisms-12-01490-s001.zip › Supplementary Table S3.pdf]

Supplementary Table S3: The amino acid identities of five conserved domains between TC-14 and HKU2-related CoVs, GS-56 and HKU24-related CoVs.

CoVs	TC-14 Amino acid identity (%)				
	3CL-pro	NiRAN	RdRp	ZBD	HEL1
OQ175205|BtRa-AlphaCoV	99.32	100	100	100	100
NC_009988|HKU2	97.29	100	99.47	100	99.67
MF094687|SADSr-CoV	97.97	100	99.64	100	100
MF094682|SADS-CoV	98.31	99.58	99.47	100	100
MF167434|PEAV	98.31	99.58	99.64	100	100
**CoVs**	**GS-56 Amino acid identity (%)**				
	3CL-pro	NiRAN	RdRp	ZBD	HEL1
MT820629|ChRtCoV HKU24	99.32	99.18	97.55	96.66	99.11
MT820630|ChRtCoV HKU24	98.3	98.36	99.02	97.89	99.71
KM349734|ChRtCoV HKU24	98.98	96.72	98.91	98.94	99.71
OQ297695|RtAp-CoV	98,64	99.59	99.35	98.94	99.41
KY370047|RtAp-CoV	97.96	98.36	99.13	98.94	99.41
