# Supplementary material for: Identification and Characterization of an Alphacoronavirus in Rhinolophus sinicus and a Betacoronavirus in Apodemus ilex in Yunnan, China
Source: Microorganisms. 2024 Jul 21;12(7):1490. doi: 10.3390/microorganisms12071490 (PMC11278907; doi:10.3390/microorganisms12071490)
Supplement: Supplementary file 1 [file microorganisms-12-01490-s001.zip › Supplementary Table S4.pdf]

Supplementary Table S4: The sequences of the degenerated sequences that were used for complete genome sequencing.

CoV	Position	Primer	Sequence (5'-3')	Fragment size (bp)
**TC-14**	1	αCoV-1F	ACATGGGGACTTAAAGATAT	583
	583	αCoV-583R	AATTAGAGGGTATAAGCCAAC	
	229	αCoV-229F	AGTGTGGTGTGTGGATATAG	2098
	2327	αCoV-2327R	ACTCAACAAATCCAGCATCC	
	1970	αCoV-1970F	GCTATGTTTAGGTCTGATGG	2360
	4330	αCoV-4330R	CAAAGACTCAGCATCCTTAC	
	3948	αCoV-3948F	TTGTGTTGATGGTATGGGTG	2183
	6131	αCoV-6131R	CCACTACGTTCAGGTATACTAG	
	5787	αCoV-5787F	CGCTTATGTGGCTTTTAGTG	1988
	7775	αCoV-7775R	CTCACACAATTCAGTAGTACG	
	7472	αCoV-7472F	GTATGCGTAGTGGTGCTAAAG	1982
	9454	αCoV-9454R	ACTCACCTGCCTTCAAAGTTC	
	9185	αCoV-9158F	GTCTTGTTGAACCATGTGTAG	1568
	10726	αCoV-10726R	AATCGTAACAGCCAAGAGTC	
	10448	αCoV-10448F	CTATGTGCACAGTCCTTTAC	1420
	11868	αCoV-11868R	TCATACTCCCACTTGACAAC	
	11572	αCoV-11572F	TGCATGGAGTATTACTGATG	1744
	13316	αCoV-13316R	CAATCACTACAATTAGGATGG	
	13009	αCoV-13009F	GGTGTTGTTGGCGTTTTAAC	1457
	14484	αCoV-14484R	ATAAGCAGTTGTAGCATCACC	
	14262	RdRp-FWD3	GGTTGGGAYTAYCCHAARTGTGA	434
		RdRp-FWD43	GAYTAYCCHAARTGTGAUMGWGC	
	14696	RdRp-RVS3	CCATCATCASWYRAATCATCATA	
	14608	αCoV-14608F	TGTTATAGGTCTAGTGCTGTTG	1755
	16363	αCoV-16363R	CATAGTTAGTGCACATAGACAC	
	16083	αCoV-16083F	ACATTGTGTTATTGGTCTAGG	1450
	17533	αCoV-17533R	AACAATCACAGACCATTTGC	
	17255	αCoV-17255F	GTAATGTTAGAGGCTGGATTG	1654
	18908	αCoV-18908R	CAAGTACACACATCCTCATC	
	18441	αCoV-18441F	CGCTAGTCTTTATAGGGCTTATG	1262
	19703	αCoV-19703R	GGCAACTTAATACTAGCACC	
	19335	αCoV-19335F	TGGTGTTCTTAAGGTTGAGG	4042
	23377	αCoV-23377R	GTGATAGTGTTGTTTGGGAC	
	20409	αCoV-20409F	TACTGATATGGTGCTTGGTC	1427
	21836	αCoV-21836R	TTACTCTCATACAGCACACC	
	23314	αCoV-23314F	GTTGAATCTCTCGTTAGTG	1344
	24658	αCoV-24658R	GTAGGTGCGTTAAAGAGGAC	

	24103	αCoV-24103F	CTATTAATGGCGCCAATATG	1687
	25790	αCoV-25790R	TTCTGTTCAACCCAATAACC	
	25464	αCoV-25464F	CTTAATGCAAGCACCAACAC	1793
	27257	αCoV-27257R	ACAGCAACATACATACGAGG	
	26950	αCoV-26950F	ACCTCTTTGAGCAATTTCTC	223
	27173	αCoV-polyA	TTTTTTTTTTTGTGTATCACTGTC	

CoV	Position	Primer	Sequence (5'-3')	Fragment size (bp)
**GS-56**	1	βCoV-1F	TGTTAGACCACCCCAGCACATG	534
	543	βCoV-543R	GGCTTGCTTTAACACAGACTCG	
	216	βCoV-216F	GCTAAGCGAGTCAACAAATAC	2209
	2425	βCoV-2425R	GAAGACAATGTAATCAGCAGC	
	2136	βCoV-2136F	GTCCAAGTTTCAGATCTTTGGC	1766
	3902	βCoV-3902R	ATCAATCCCACACTTCAAGC	
	3545	βCoV-3545F	CTATGTTGCAGCTTATGAGG	2133
	5658	βCoV-5658R	CACTTGACAAATCTGCTTGG	
	5217	βCoV-5271F	GCAGGCAAAGTACTCTATCAG	1933
	7204	βCoV-7204R	CCAATACCTGACACGTCTATG	
	6888	βCoV-6888F	TGGTTGAAGAAGCCAAGTGAC	1705
	8593	βCoV-8593R	CCAGTCTTCACACATGCTTTC	
	8274	βCoV-8274F	ACATTTATTGGCTGTGCACG	1849
	10123	βCoV-10123R	TGGTAGAGCACATCATTACC	
	9842	βCoV-9842F	CTGGGTATTTAGTTACTTCAGG	1542
	11384	βCoV-11384R	AACAGAAGGCACCACATATG	
	11065	βCoV-11065F	TGCAATCAAAACGCACTAGAG	1597
	12662	βCoV-12662R	CCACACATTACCAGCATATG	
	12370	βCoV-12370F	GTAAGCTTGAACGTATGGCTG	1444
	13814	βCoV-13814R	CGTATATATCAAGGCCAGTTC	
	13476	βCoV-13476F	CTGTGTAGGTTCAAGTGCTAC	1371
	14847	βCoV-14847R	ACAGAACTACCTTCCTTAAGC	
	14594	βCoV-14594F	GGTGTTGTTATGAATATGGATG	1073
	15667	βCoV-15667R	GACATCAAGGCACATACATTG	
	15427	RdRp-FWD3	GGTTGGGAYTAYCCHAARTGTGA	327
		RdRp-FWD43	GAYTAYCCHAARTGTGAUMGWGC	
	15754	RdRp-RVS3	CCATCATCASWYRAATCATCATA	
	15523	βCoV-15523F	AGTCTTGAGTGAGATTGTTATG	1304
	16826	βCoV-16826R	TTCACGATCGCTTACTATCTC	
	16693	βCoV-16693F	GGACTGATGTGGACGATTAC	1396
	18089	βCoV-18089R	CTGCATATTACTCATAACGC	
	17803	βCoV-17803F	TTCGAGTGCTGTCAATATGC	1503
	19306	βCoV-19306R	CTTATCCACATTGCAGTTCC	
	19042	βCoV-19042F	CAATTGGAACCTTGAGTATCC	1525
	20567	βCoV-20567R	ACCCACCAATTATCTTCTGG	
	20282	βCoV-20282F	GTTATCTTCAGCCAGTTAACC	2425
	22707	βCoV-22707R	TGAGAAACAACATTGATGGG	
	22374	βCoV-22374F	AGTGCTATAATGGGCTATTG	1662
	24036	βCoV-24036R	TACTCCTTGCGAAACATCTAC	
	23732	βCoV-23732F	CCATTTGTAATTATGATCCGC	1482
	25214	βCoV-25214R	CTAGGATTCTTCTTGGTAAC	

	24938	βCoV-24938F	AGTACCTTCCCCAATCTCATG	1456
	26394	βCoV-26394R	TCTCTACAAGCCGCATAGTC	
	26130	βCoV-26130F	AGCTGTTGGTGTTTGTAATC	1690
	27820	βCoV-27820R	GCCATTTTACATACATCTCG	
	27467	βCoV-27467F	TAGCACCTAAGAGTGGATAC	2023
	29499	βCoV-29499R	TCCTCAATTATGGGCCTAAC	
	29195	βCoV-29195F	CTACAGTTTGGTTACACGAG	2006
	31201	βCoV-31201R	GTGACAAGACATCCATTCTG	
	30900	βCoV-30900F	GTAAGCAGAAGAAAGGGCAAG	371
	31271	βCoV-polyA	TTTTTTTTTTTTGTGATTCTTCCAATTGG	
